# Seroprevalence of *Neospora caninum* Infection and Associated Risk Factors in Cattle of Sistan Areas, Southeastern Iran in 2016

**Published:** 2019

**Authors:** Marzieh NOORI, Mehdi RASEKH, Maryam GANJALI, Saeid Reza NOUROLLAHI FARD

**Affiliations:** 1. Department of Veterinary Medicine, Faculty of Veterinary Medicine, University of Zabol, Zabol, Iran; 2. Department of Clinical Sciences, Faculty of Veterinary Medicine, University of Zabol, Zabol, Iran; 3. Department of Parasitology, Faculty of Veterinary Medicine, University of Zabol, Zabol, Iran; 4. Department of Pathobiology, School of Veterinary Medicine, Shahid Bahonar University of Kerman, Kerman, Iran

**Keywords:** Cattle, ELISA, Neospora caninum, Iran

## Abstract

**Background:**

*Neospora caninum* is protozoan parasitic disease now described as the major cause of abortion and other reproductive issues. The aim of this study was to determine the seroprevalence of *N. caninum* in cattle breeds of the Sistan region, southeastern border area of Iran.

**Methods:**

Using an ELISA kit (ID.VET, France), the antibodies against *N. caninum* in cattle of Sistan was evaluated in 2016. Overall, 184 blood samples from apparently healthy cattle in the eastern border area of Iran Sistan were collected for assessment of antibodies against *N. caninum*. The values greater than or equal to 50%, were considered positive based on manufacture’s manual for ELASA kit.

**Results:**

3.8% of 184 cattle have antibody against *N. caninum*. Chi-square test showed that the seroprevalence among Holsteins, Sistan and cross-breed was 9.4%, 0%, and 4.3%, respectively. No significant difference was observed among the breeds (*P*>0.05). The seroprevalence was decreased as the age of cow increased and there is no significant difference between the prevalence of *N. caninum* and different city area. No statistically significant relationship between the seroprevalence of *N. caninum* and history of abortion, lactation number and infertility was observed. Although there was no significant difference between the cattle breeds of the Sistan region all the Sistani cows were negative for the antibody against *N. caninum.*

**Conclusion:**

Infection rate with *N. caninum* in bovine population in Sistan region is very low. Environmental and management factors are the major causes, which influence the regional prevalence.

## Introduction

Neospora is a protozoan of the family Sarcocystidae in the phylum of Apicomplexa. *Neospora caninum* was first detected in a dog in 1984 with the signs of myositis, lameness and encephalitis (1). *N. caninum* is distributed worldwide and its association with the abortion has risen as a concern for the cattle operation industry (2). The whole range of mammals including cattle, sheep, goats, horses, rhinos are intermediate hosts for this parasite but it is mainly important in cattle and dog. Canids are the definitive host and excreting oocysts in their feces is a prominent risk factor for the occurrence of neosporosis in cattle (3). The seroprevalence rate of infection in dogs was reported 0% to 67.6% and 10.6% to 33% from worldwide and Iran, respectively (4–7). Two ways of transmission are mainly recognized; intake of contaminated food by oocysts (horizontal) and transplacental transmission (vertical) (3, 8).

*N. caninum* is associated with the reproductive disorders in cattle. The economic losses due to the infection is considerably high and it is observed as abortions, stillbirth, calves mortality, increased calving intervals, culling the infected cows and high expenses of diagnosis and treatment of suspected animals (9–11). The autolysis or mummification of fetus occurs during pregnancy (12). In California, the economic losses caused by neosporosis have been estimated at around $ 35 million (4, 5). The serological tests are extensively used for the detection of anti-*N. caninum* antibodies, including ELISA, the direct agglutination test (DAT), the indirect fluorescent antibody technique (IFAT) and immunoblots (13).

Infection with *N. caninum* has been reported from many parts of the world (14). The prevalence of the disease in cattle population of Iran based on different studies has been reported 38.8%, 38.5%, 21%, 17.4%, 15.18 %, 12.6% and 7.8% in Tehran, Garmsar, Ahvaz, Hamedan, Mashhad, Kerman and Kurdistan, respectively (3, 15–20). However, there is no report available for the detection of antibody against the organism in Sistan region (eastern border area of Iran) and its related risk factors. Furthermore, cattle populations of Sistan are including different important breeds like Holstein, Sistani (derived from Zebu) and cross breeds.

We aimed to determine the seroprevalence of *N. caninum* in cattle breeds of the Sistan region, southeasthern border area of Iran.

## Materials and Methods

Blood samples from 184 cattle over 6 months were collected in 2016, 10 ml fresh blood from the jugular vein was taken and was transferred to a tube containing pro-coagulation factor. The Blood samples were transferred immediately to the laboratory of Parasitology, Faculty of Veterinary Medicine of University of Zabol, Iran. Blood samples were centrifuged at 3,000 rpm for 10 min and the serum of each was isolated. The isolated serums were stored at −20 °C until the ELISA test. The detection of antibodies against *N. caninum* was carried out using an ELISA kit. For this purpose, commercially available kit (ID Screen *N. caninum* indirect multi-species, IDVet, France) were used. Reading the OD of samples was performed using Elisa reader (Anthos 2020 Austria). The presence or absence (being negative or positive) of antibodies was determined based on the ratio of positive cases (s/p)% by following formula:

$$S/P(\%)=\frac{{\mathrm{OD}}_{\mathrm{Sample}}-{\mathrm{OD}}_{\mathrm{Negative}}}{{\mathrm{OD}}_{\mathrm{Positive}}-{\mathrm{OD}}_{\mathrm{Negative}}}\times 100$$

Values greater than or equal to 50%, between 40% and 50% and equal to or less than 40% were considered positive, suspected and negative, respectively.

Statistical relationship between antibody against *N. caninum* and age, breed, parities number, history of abortion, reproductive (pregnancy) status and the city area with the chi-square test and Fisher’s exact test were analyzed. SPSS software (ver. 18, Chicago, IL, USA) was used for statistical analysis. The level of *P*-value<0.05 was considered significant.

## Results

Based on the results of the ELISA test, anti-body against *N. caninum* were found in 7 of the 184 (3.8%) sera. As shown in Table 1, there was no significant correlation between seropositivity and breeds (*P*>0.05).

**Table 1:** Seroprevalence of *N. caninum* in relation to breed, city (area), age, pregnancy status, history of abortion, number of parity and reproductive status

***Variable***		***The number of tested animals***	***The number of positive cases***	***Seroprevalence (%)***
Breed	Holstein	102	5	4.9
	Sistani	23	0	0
	cross-breed	59	2	3.4
City(area)	Zahak	57	5	8.8
	Hirmand	24	0	0.0
	Zabol	23	0	0.0
	Nimrouz	21	1	4.8
	Hamoon	59	1	1.7
Age	< 2 years	22	3	13.6
	2< years<4	54	2	3.7
	4<years<6	47	2	3.4
	≥ 6 years	61	0	0.0
Pregnancy status	Pregnant	28	2	7.1
	Nonpregnant	134	2	1.5
History of abortion	Yes	11	1	9.1
	No	151	3	2.0
Number of parity(ies)	0	15	1	6.7
	1<parity<4	117	3	2.6
	>4	30	0	0
Reproductive status	Fertile	150	3	2.0
	Infertile	12	1	8.3

Furthermore, there was no significant relationship between the history of abortion and seroprevalence of *Neospora* in cattle. There was no statistical relationship between seroprevalence and number of calving, as well as between seroprevalence of neosporosis and cattle fertility.

## Discussion

The seroprevalence of neosporosis was reported 0.7%–97.2% in cattle (4, 5). Infection with *N. caninum* has been reported from several countries of the world like Peru, Greece, Spain, Ireland, Brazil, Canada, Australia, and Korea (4, 5). Recently, it has also been reported from different parts of Iran such as Tehran, Garmsar, Ahvaz, Hamedan, Mashhad, Kerman and Kurdistan (3, 15–20).

In the present study, antibodies against *N. caninum* were detected in 3.8% of cows and it was the least reported regional seroprevalence of the disease in Iran. The results of the researches indicate a different outbreak in different regions. The exact cause of this finding is unknown, but the dogs associated with the farms, study design, climatic variations and the method of diagnosis are influential factors (3). As the climate condition of Sistan region is warm and dry in the first half and cold and dry in the second half of the year and because the presence of parasite oocysts excreted by the final hosts in the environment, highly depends on temperature and other environmental factors, the environmental condition of this region is not favorable for viability of organism (3).

ELISA and IFAT are the main serological tests extensively used for the detection of *N. caninum* antibodies (13, 21). Compared with ELISA, the IFAT is time-consuming and more expensive. Furthermore, the sensitivity and specificity of ELISA test is considerably high, ≥95% and ≥97%, respectively. The mentioned advantages made the ELISA as an authentic test for detecting the infected animals and herds to *N. caninum.* Todays, many laboratories in Canada use the ELISA test as the test of choice for detecting antibody against *N. caninum* (21).

Abortion is the major cause of economic and reproductive loss of Iran’s cattle farms due to neosporosis. In most studies, seroprevalence of neosporosis has been correlated with abortion in cattle. In general, the abortion rate in infected animals is three to seven times higher than uninfected animals (13, 22, 23). In Garmsar, Iran, 95% of aborted cattle have anti-body against *N. caninum* in their serum (17). In present study, although the abortion rate in seropositive cows was more than seronegative ones this correlation was not statistically significant. This finding is inconsistent with the research performed in Kurdistan province, Iran (16). The association between seropositivity and abortion may vary considerably by different serological assays or different cutoffs values (24, 25). Furthermore, the small number of cattle with a history of abortion could also be as a presumptive cause of the present finding (17).

In this study, the relationship between seroprevalence of *N. caninum* and the age of animal was evaluated. Seroprevalence of *N. caninum* decreased significantly by increasing the age of cows (*P*=0.014). There has been no consistent finding on the relationship between age and seroprevalence of the disease until now. Some studies found no relationship between the age and seroprevalence (5, 15, 19, 26), while the prevalence of the disease increases with the aging (3, 27). However, the prevalence of *N. caninum* in cattle farms with high average age is low because most infected animals are in latent stages and are less likely to test positive (21). Younger cows had higher CI-ELISA inhibition percentage values than older cows (28). Infection of fetuses is due to infected cows and if these calves have not been infected again, the titers of antibody subside over time, resulting in decrease in seroprevalence with cow aging (28). In our study, the calves might be infected in their fetal period and the antibody was detectable after the birth but consequently declined over time with no exposure.

In the present study, the seroprevalence of *N. caninum* infection in Holstein cows were more than other breeds. The interesting finding of this study was for the Sistani breeds cows so that all of 23 cows of Sistani breed were seronegative for the *N. caninum.* Most researchers have found no correlation between the seroprevalence of *N. caninum* and breed of the cow (19) but some researchers have found the differences between breeds. In Brazil and in Iran, at the Holstein cows are more susceptible to provoke antibody against *N. caninum* in comparison to cross-breeds (15, 29); however, the less sensitivity of Holsteins compared to jersey and Jersey/Holstein cows is also reported (30) but this finding might be related to the management factors other than the breed susceptibility.

Dogs are the definitive hosts for *N. caninum,* which excreted oocysts in their feces. Shedding oocysts in the environment is an important risk factor for the occurrence of neosporosis in cattle (31, 32). As the infection rate with *N. caninum* increases in dogs, the higher rate of infected cows could be expected. The close contact with infected uterine discharges of the cattle increases the seroprevalence of neosporosis in dogs (33, 34). Furthermore, suppression of immune system of dogs caused by Canine visceral leishmaniasis (CVL) can increase dog’s susceptibility to *N. caninum* infection (35). In a study performed in the CVL endemic areas Northwestern Iran, the co-infection of domestic dogs with *N. caninum* and *L. infantum* is common. The infection of dogs with *L. infantum* increases susceptibility to *N. caninum* infection (36).

The proximity of infected dog to cattle farms is a prominent risk factor; dogs kept in farms or their neighborhood may pose an infection risk (3). Thus, the type of cattle husbandry systems and their management strategies can also influence the prevalence of neosporosis in a certain operating system. In the rural areas, dogs are in near contacts to the cows and eventually, the risk of exposure will increases. On the other hand, the risk of infection may increase in industrial farms when a dog is freely passing through the animals.

## Conclusion

The prevalence of neosporosis in cows of southeastern border of Iran is low (3.8%). Therefore, infection with *N. caninum* is not one of the major causes of cattle abortion in this region. Further comprehensive studies and investigations are recommended to determine the contribution and the portion of *N. caninum* to cattle abortion among other infectious and non-infectious agents causing abortion in cows of Sistan.
